# Anxiety among children a year after the onset of the COVID-19 pandemic: a Brazilian cross-sectional online survey

**DOI:** 10.3389/fpubh.2024.1372853

**Published:** 2024-06-19

**Authors:** Marla Andréia Garcia de Avila, Tainara de Jesus Amorin, Pedro Tadao Hamamoto Filho, Graziela Maria Ferraz de Almeida, Patricia Olaya-Contreras, Malin Berghammer, Margaretha Jenholt Nolbris, Stefan Nilsson

**Affiliations:** ^1^Department of Nursing, Botucatu Medical School–UNESP-São Paulo State University, Botucatu, Brazil; ^2^Department of Neurology, Botucatu Medical School–UNESP-São Paulo State University, Botucatu, Brazil; ^3^Institute of Health and Care Sciences, Sahlgrenska Academy, University of Gothenburg, Gothenburg, Sweden; ^4^Centre for Person-Centred Care, Sahlgrenska Academy, University of Gothenburg, Gothenburg, Sweden; ^5^Department of Health Sciences, University West, Trollhättan, Sweden; ^6^The Queen Silvia Children’s Hospital, Gothenburg, Sweden

**Keywords:** anxiety, children, COVID-19, pandemics, pediatric nursing

## Abstract

**Aim:**

This study seeks to build upon a prior investigation into the impact of the COVID-19 pandemic and to evaluate the prevalence of anxiety among Brazilian children, along with its associated factors, one year after the commencement of the pandemic.

**Design:**

A cross-sectional study.

**Methods:**

A survey was conducted from April–May 2021 in Brazil. Children aged 6–12 and their guardians from five Brazilian regions were included. The Children’s Anxiety Questionnaire (CAQ; scores 4–12) and Numerical Rating Scale (NRS; scores 0–10) were used to measure anxiety.

**Results:**

Of the 906 children, 53.3% were girls (average age = 8.79 ± 2.05 years). Mothers responded for 87.1% of the children, and 70.9% were from the Southeast region. Based on a CAQ score of ≥9 and an NRS score of ≥8, the anxiety prevalence was 24.9 and 34.9%, respectively. Using logistic regression, a CAQ ≥9 score was associated with older children and children with chronic disease or disability. An NRS score of ≥8 was associated with reduced family income during the pandemic, the person caring for the children, and with children with chronic disease or disability.

**Conclusion:**

These findings suggest the need to implement public health actions aimed at children with chronic diseases and disabilities and their parents to guide them regarding the warning signs and negative emotions. This study contributes to characterizing the evolution of the pandemic in Brazil and provides a basis for comparison with the literature from other countries.

## Introduction

Owing to the COVID-19 pandemic, children and their guardians experienced extremely challenging and stressful situations such as a sudden change of routine, lack of in-person classes, reduced family budget, and social and familial distancing (1). The most prevalent measure to contain the contagion of the COVID-19 pandemic was social distancing. Hence, schools were closed during the first year of the pandemic in Brazil, which affected more than 35.2 million children and adolescents (approximately 17% of the nation’s population)(2) compared with Sweden, where preschools and primary schools have remained open throughout the pandemic to prevent adverse effects such as loss of learning opportunities and a negative impact on children’s mental and physical health (3).

Brazil has deep social inequalities. Children depend on public schooling, especially those living in highly vulnerable conditions, and the closure of on-site schools resulted in increased hunger and nutritional deficiencies in the absence of school meals, which increased violence. A narrative synthesis of reports from the first wave of the COVID-19 pandemic (February–July 2020) with 36 studies from 11 countries, which involved 79,781 children and adolescents and 18,028 parents, showed that short-term school closures, which were part of the social lockdown measures, resulted in adverse mental health symptoms and health behaviors among children and adolescents (4).

A global meta-analysis of 74 studies from 2023, which included 478,882 participants (mean age = 13.4 years, 52.3% female) shows the pooled rate of children and adolescents fulfilling diagnostic criteria for anxiety disorders was 13.0% (95% confidence intervals (CI) =4.9–30.1); the pooled prevalence of anxiety symptoms was 26.5% (95%CI = 20.3–33.9). Anxiety symptoms were significantly more prevalent in females than males (*B* = 0.103, *p* < 0.001), significantly higher during the second wave of COVID-19, following July 2020, than during the first wave, prior to June 2020, (*Q* = 8.136, *p* = 0.017), and during school closure (*Q* = 8.100, *p* = 0.014) (5). Another meta-analysis of 191 studies, which included 1,389,447 children and adolescents, indicated that the pooled prevalence of depressive symptoms from 129 studies (*n* = 524,417) was 31% (95% CI 27–35%); and the prevalence of mild, moderate, and severe depressive symptoms was 19% (95% CI 15–24%), 13% (95% CI 10–16%), and 6% (95% CI 4–9%), respectively. The pooled prevalence of sleep disturbances from 50 studies (*n* = 104,219) was 42% (95% CI 33–52%) (6). Age, grade levels, education levels, gender, geographical regions, and electronics use were associated with an increased prevalence of mental health symptoms. The prevalence of mental health symptoms also increased as the pandemic progressed, although signs of recovery and stabilization were observed (6).

The present study, serving as a follow-up to the sole investigation conducted in Brazil, is not primarily aimed at statistical comparison between these time points. However, the antecedent study, included in a meta-analysis, revealed that the prevalence of anxiety among 289 Brazilian children aged 6–12 years at the onset of the pandemic was 19.4% (*n* = 56) based on the Children’s Anxiety Questionnaire (CAQ) and 21.8% (*n* = 63) according to the Numerical Rating Scale (NRS). Higher levels of anxiety were associated with social distancing, identified primarily in people who lived together and had a lower level of education as reported by guardians, and children whose guardians were younger. Lower levels of anxiety were associated with social distancing between children and their parents (7). Similar research with 774 Swedish children (6–14 years old) during the first wave of the COVID-19 pandemic showed that the prevalence of children with intense anxiety (CAQ score ≥ 9 or NRS score > 7) in the total study population was 2.5% (CAQ score), and 2.7% experienced high levels of anxiety, which is in contrast with many other studies. Maintaining a normal life could be critical in preventing higher anxiety and depression levels in children during a pandemic (3).

The COVID-19 pandemic has lasted longer than many of us expected. Researchers considered that more studies on the 2nd and 3rd waves of COVID-19 are needed to evaluate the long-term impact of COVID-19 on the mental health of young people (5).

This study seeks to build upon a prior investigation into the impact of the COVID-19 pandemic and to evaluate the prevalence of anxiety among Brazilian children, along with its associated factors, one year after the commencement of the pandemic.

## Methods

### Study design

A survey cross-sectional study was conducted between April 20 and May 31, 2021. Data communication follows the recommendations of the Strengthening the Reporting of Observational Studies in Epidemiology STROBE statement (8) to ensure the study method quality Supplementary file 1.

### Sample and data collection

Residents (adults aged 18 years and older) across five regions in Brazil (North, Northeast, Midwest, Southeast, and South) were contacted to participate in this study. To reach all potential participants, we conducted an online survey using Google Forms during the period between April 20 and May 31, 2021. According to the Oxford Stringency Index for the survey period, in Brazil, the reported confirmed COVID-19-related deaths were 171.26 per 100,000 population on April 20, 2021, and 213.20 per 100,000 population on May 31, 2021. During the data collection period, school disclosure was categorized as level 3, meaning it was required at certain levels (9).

Brazil has around 27,424,401 children between 5 to 14 years old (10). Consequently, we employed a non-probability sampling method, specifically convenience sampling, to gather all the necessary information (11). The online survey was disseminated by four researchers and six collaborators (master’s and doctoral student nurses) through social media platforms (Facebook and Instagram) as well as personal communication channels (WhatsApp and email). We provided guardians in these regions with a concise overview of the study’s objectives and instructions on how to participate, complete the forms, and conduct interviews with their children.

The survey had 24 closed items. The variables measured for the children included gender (female, male, or non-binary), age, school status (vacation/not going to school, home school, online/home class, hybrid class, presential class), who took care of the children (father, mother, both, or other), if guardians were health professionals during COVID-19 (yes or no), if they had a chronic disease or disability (yes or no), how many adults and children lived in the same house, whether there were suspected or confirmed diagnoses of COVID-19 among immediate family members (yes or no), how long the children had been social distancing, type of dwelling (house or apartment), region of residence (North, Northeast, Midwest, South, and Southeast), and individual response to the NRS and CAQ questionnaire. Owing to the high number of cases and deaths caused by the COVID-19 pandemic in Brazil, presential classes were not mandatory for children during the data collection period. The school status were: home school, hybrid classes, presential school, on vacation or missing school. The quantitative variables for the guardians included gender (female, male, or other), age, relationship with the child (mother/stepmother, father/stepfather, grandmother/grandfather, and others), schooling (elementary school, high school, college, or postgraduate degree), income reduced during the pandemic (yes or no), and their perceptions of their child’s understanding of the pandemic (a lot, some, a little, or nothing). We followed the design used in a similar previous study, which assessed anxiety at the beginning of the pandemic between 25 April and 25 May, 2020 (7).

### Participants

The inclusion criteria for enrolment included Brazilian school children aged 6–12 years and their guardians from all five regions in Brazil: North, Northeast, Midwest, Southeast, and South. The exclusion criteria encompassed guardians aged under 18 years and those lacking access to computers, telephones, and Wi-Fi and skills necessary to read and respond.

### Assessments of anxiety

The CAQ and NRS were used to measure anxiety in the children. The CAQ, developed in Sweden is available in Arabic, English, Swedish, (12, 13), and Portuguese (14). The CAQ contains four items with four images of facial expressions and three response options; each represents different levels of emotional intensity (12, 13). Children responded based on the four facial expressions, one at a time, and choose between the three responses (i.e., a little = 1, some = 2, and a lot = 3). The faces of happy/content and calm/relaxed were measured as 3–2–1, and those of tense/nervous and worried/afraid were measured as 1–2–3. The minimum aggregate score was 4, representing the lowest anxiety level for all four items. The CAQ in Brazilian Portuguese was recently validated and demonstrated satisfactory results among professionals and children (14). The CAQ was based on the State-Trait Anxiety Inventory (15) and has previously shown construct validity in conjunction with outpatient surgery (16).

The NRS, an 11-point scale, is scored from 0–10. It has been validated for the evaluation of pain intensity in children (17) and unpleasantness. However, there is no consensus on the NRS anchors for measuring unpleasantness in children (18). In this study, anxiety was assessed using the NRS, where 0 was equivalent to “calm,” and 10 meant “very anxious.” The NRS was easy to administer and demonstrated good evidence for its construct validity (17, 18).

Following previous research, (7) the CAQ cut-off value was set at the level [low <9 or high ≥9]; scores of 9 and higher indicated intense anxiety. For the NRS, the cut-off value was set at the level [low <8 or high ≥8]; thus, scores of 8 or higher indicated intense anxiety.

### Statistical analysis

The Shapiro–Wilk test was used to evaluate the distribution of continuous data. Comparisons of continuous data between the groups were performed using the Mann–Whitney U-test for unpaired data that are not normally distributed. Kruskal–Wallis and Dunn’s or ANOVA tests were performed for multiple-group comparisons. Spearman’s rank (rho) correlation coefficients were calculated between the CAQ and NRS scores. A chi-square test, as a two-tailed test (n > 30), and Fisher’s exact test were used to compare the proportions in the different groups. Odds ratios (ORs) were calculated to evaluate the association between the outcome variables, such as the dependent variable (NRS or CAQ, as binary categories defined [</>]). For the CAQ, a score higher than or lower than the mean value, and a single standard deviation, resulted in the cut-off value as lower or higher than 9. In addition, scores of 9 and higher indicated intense anxiety. For the NRS, the cut-off value was set at the 8 and higher.

A logistic regression was performed to evaluate the associations between the dependent variable (i.e., high or low anxiety scores of the CAQ (≥ 9) and NRS (≥8), respectively) and the independent variables. The presence of anxiety was defined via the cut-off values of ≥9 and ≥ 8 for the CAQ and the NRS, respectively. For the analysis, we considered the children’s anxiety as the dependent variable. Independent variables were age, gender, decreased income during the pandemic, presence of a chronic disease or disability, the region in Brazil, and who takes care of the child. The child’s perceived comprehension of the situation was considered a potential confounder.

For all tests, the level of statistical significance was set at 5%. Statistical analyses were performed using IBM SPSS Statistics for MacBook, version 24 (IBM Corp., Armonk, NY, USA). Finally, the point prevalence of anxiety was compared with a previous survey via CI set at 95% and the respective ORs. For all tests, the level of statistical significance was set at 5%.

### Ethical information

This study was approved by the Research Ethics Committee of Brazil (CAAE: 30547320.0.0000.0008 and Opinion n° 4.128.847) and a new application and approval were granted for data collection a year after the pandemic (Opinion n° 4.593.273). We obtained approval for data collection using the same design as that of the first study. The guardians and children agreed to participate in the study through an electronic register.

## Results

A total of 920 respondents accessed the data collection instrument. Of these, ten refused to participate, and four were excluded as they did not meet the inclusion criteria (see above). Hence, 98.5% (*n* = 906) of children and their guardians participated in the study (Supplementary file 2).

Among 906 children, 53.3% were girls (average age = 8.79 ± 2.05 years), 70.9% were from the Southeast region, 67% (*n* = 607) studied in private schools and 10.6% (*n* = 96) had chronic diseases or disabilities. Based on a CAQ score of ≥9 and an NRS score of ≥8, the anxiety prevalence was 24.9 and 34.9%, respectively. The most common chronic disease was asthma/bronchitis (48.0%, *n* = 46) and the most common disability was attention deficit hyperactivity disorder (ADHD; 8.3%, *n* = 8).

The guardians’ mean age was 38.45 ± 8.07 years. Most guardians (87.1%; *n* = 789) were mothers/stepmothers, approximately 49.6% (*n* = 449) went to graduate school, and 70.9% (*n* = 642) were from the Southeast region. Among the participants, 47.7% (*n* = 432) had income reduction during the first year of the COVID-19 pandemic, 77.7% (*n* = 704) lived in a house, 52.1% (*n* = 472) suspected COVID-19 among family members, and 26.5% (*n* = 240) received positive confirmation regarding the same. A total of 45.9% (*n* = 416) of the guardians worked from home, and 22.5% (*n* = 204) were healthcare and frontline professionals during the COVID-19 pandemic (Supplementary file 3).

Table 1 shows the significant difference in the prevalence of anxiety based on the CAQ ≥ 9 and NRS ≥8 scores at different ages. There were statistical differences between the prevalence of anxiety for CAQ (*p* = 0.028), but not for NRS (*p* = 0.415).

**Table 1:** Summary of the prevalence (%) and [95%CI] of anxiety (CAQ score ≥ 9; NRS score ≥ 8) for children by age, *n* = 906, Botucatu, SP, Brazil, 2021.

Age (years)	Total (*n*)	CAQ score ≥ 9^1^	NRS score ≥ 8^2^
6	176	31 (17.6) [12.7–23.9]	63 (35.8) [29.1–43.1]
7	132	29 (22.0) [15.8–29.8]	50 (37.9) [30.1–46.4]
8	112	23 (20.5) [14.1–28.9]	34 (30.4) [22.6–39.4]
9	136	38 (27.9) [21.1–36.0]	56 (41.2) [33.3–49.6]
10	113	32 (28.3) [20.8–37.2]	37 (32.7) [24.8–41.8]
11	124	34 (27.4) [20.3–35.9]	36 (29.0) [21.8–37.6]
12	113	39 (34.5) [26.4–43.7]	40 (35.4) [27.2–44.6]
Total	906	226 (24.9) [22.2–27.9]	316 (34.9) [31.9–38.0]

CAQ, children’s anxiety questionnaire; NRS, numerical rating scale.

1 Chi-square test, *p* = 0.028.

2 Fisher’s exact test, *p* = 0.415.

Table 2 shows the prevalence of anxiety according to both instruments and their associations with each variable. A chronic disease or disability influenced children’s higher levels of anxiety, 40.6% (*n* = 39) in the CAQ ≥ 9 (*p* < 0.001) and 49% (*n* = 47) in the NRS ≥8 (*p* = 0.002).

**Table 2:** Comparison of the prevalence of anxiety among children (CAQ Score ≥ 9; NRS Score ≥ 8) based on the main characteristics of the population, *n* = 906, Botucatu, SP, Brazil, 2021.

	CAQ ≥ 9	%	*p* ^1^	NRS ≥8	%	*p* ^2^
*Gender*						
Girls (*n* = 483)	128	26.5	0.256	170	35.2	0.961
Boys (*n* = 422)	98	23.2		147	34.8	
*Decreased income during pandemic*						
Yes (*n* = 432)	119	27.5	0.084	176	40.7	<0.001
No (*n* = 474)	107	22.6		140	29.5	
Chronic disease or disability*						
Yes (*n* = 96)	39	40.6	<0.001	47	49.0	0.002
No (*n* = 810)	187	23.1		269	33.2	
*Region in Brazil*						
North (*n* = 46)	11	23.9	0.982	13	28.3	0.004
Northeast (*n* = 92)	24	26.1		21	22.8	
Midwest (*n* = 46)	11	23.9		13	28.3	
South (*n* = 80)	18	22.5		20	25.0	
Southeast (*n* = 642)	162	25.2		249	38.8	
*Take care of the children*						
Both parents (*n* = 207)	48	23.2		52	25.1	
Mother (*n* = 466)	120	25.8		167	35.8	
Father (*n* = 55)	12	21.8	0.902	24	43.6	0.007
Other relative (*n* = 127)	32	25.2		50	39.4	
Baby sister (*n* = 47)	13	27.7		21	44.7	
Alone (*n* = 04)	01	25.0		02	50.0	
*Child’s perceived comprehension*						
None (*n* = 10)	3	30.0	0.594	4	40.0	0.001
A little (*n* = 154)	41	26.6		45	29.2	
Some (*n* = 230)	63	27.4		104	45.2	
A lot (*n* = 512)	119	23.2		163	31.8	

CAQ, children’s anxiety questionnaire; NRS, numerical rating scale.

1 Chi-square test.

2 Fisher’s exact test; significant at *p* < 0.05.

Tables 3, 4 present the binary logistic regression results for the CAQ and NRS, with the independent variables included. Children with chronic illnesses or disabilities had higher CAQ (*p* < 0.001) and NRS (*p* = 0.010) scores and were likely to exhibit anxiety than those without them (CAQ: OR = 1.1 CI 1.067–1.24, Table 3; NRS: OR = 1.1 CI 1.067–1.24; Table 4).

**Table 3:** Logistic regression for the CAQ: anxiety (≥ 9) or Not (< 9), *n* = 906, Botucatu, SP, Brazil, 2021.

Variable	B	S.E.	Wald	df	Sig.	OR	95% CI for OR
Child’s age	0.140	0.038	13.426	1	**<0.001**	1.1	1.0–1.2
Chronic disease or disability	0.874	0.227	14.816	1	**<0.001**	2.3	1.5–3.7

CAQ, children’s anxiety questionnaire; CI, confidence interval; SE, standard error; OR, odds ratio.

**Table 4:** Logistic regression for the NRS ≥ 8, *n* = 906, Botucatu, SP, Brazil, 2021.

Variable	B	S.E.	Wald	df	Sig.	OR	95% CI for OR
Chronic disease or disability	0.685	0.225	9.249	1	**0.002**	1.9	1.2-3.0
Decreased income during pandemic*	0.483	0.145	11.033	1	**0.001**	1.6	1.2–2.1
Region in Brazil**			11.786	4	**0.019**		
Northeast	−0.499	0.345	2.095	1	0.148	0.6	0.3–1.1
Midwest	−0.657	0.266	6.092	1	**0.014**	0.5	0.3–0.8
South	−0.301	0.346	0.755	1	0.385	0.7	0.3–1.4
Southeast	−0.623	0.275	5.130	1	**0.024**	0.5	0.3–0.9
Take care of the child†			11.494	4	**0.022**		
Only mother	0.436	0.193	5.123	1	**0.024**	1.5	1.0–2.2
Only father	0.840	0.324	6.723	1	**0.010**	2.3	1.2–4.3
Other relative	0.589	0.248	5.624	1	**0.018**	1.8	1.1–2.9
Baby-sitter	0.821	0.340	5.851	1	**0.016**	2.2	1.1–4.4
Child’s perceived comprehension‡	0.292	0.192	2.309	1	0.129	1.3	0.9–1.9

CI, confidence interval; NRS, numerical rating scale; SE, standard error; OR, odds ratio.

* Reference category: no.

** Reference category: North.

† Reference category: both parents.

‡ Reference category: No/some.

Table 3 shows that the CAQ scores were also influenced by the child’s age (*p* < 0.001). The children’s age was associated with the presence of anxiety. Older children were more likely to be anxious (OR = 1.1 CI 1.067–1.24).

Table 4 shows significant associations between anxiety and having a chronic illness or disability, decreased parental income during the pandemic, the children’s caregiver, and the region where the child lives. The children whose parents reported decreased income during the pandemic were more likely to exhibit anxiety than those whose parents did not (OR = 1.6 CI 1.5–1.2). In addition, compared with the children cared for by both mother and father, those cared for by a single parent, another relative, or a babysitter were more likely to exhibit anxiety.

## Discussion

Different factors influenced the level of anxiety. According to the CAQ ≥ 9 (*p* < 0.001) scores, children’s age was associated with higher anxiety levels. The prevalence of anxiety was higher for 12-year-olds based on the CAQ ≥ 9 scores (39%, *n* = 113) and for 6-year-olds based on the NRS ≥8 scores (63%, *n* = 31). These differences may be due to the characteristics of the instruments. Conversely, the CAQ considers different feelings in measuring a child’s anxiety than the NRS, which can be easier for young children.

The person who takes care of the children was another factor associated with a higher prevalence of anxiety. Children were less likely to experience anxiety when both parents cared for them, based on the NRS ≥8 scores. At the beginning of the COVID-19 pandemic, previous research showed that children who were keeping social distance together with their mothers and fathers had lower CAQ scores than those who were isolated with someone other than their parents (7). A systematic review highlighted the influence of the family relationships associated with mental health changes among children and adolescents during the pandemic. In several cases, parents and children reported that the pandemic had encouraged higher levels of family intimacy (19).

There was an association between the reduction in income among Brazilian families during the first year of the COVID-19 pandemic and the prevalence of related anxiety according to the NRS, which indicated the pandemic’s adverse a negative influence on families. Social distancing affected income from tourism and various services, such as clothing, toys, and home appliance stores, which affected daily consumption, purchase of medications, paying household bills, lower hiring by Brazilian commerce, and even the payment of health plans (20). Economic instability tended to increase anxiety levels among the population, as suggested by a study with 2,510 adults at the Brazilian borders, where the prevalence of anxiety was 63.5% during the pandemic. (21). We did not investigate whether an association exists between family income and children’s anxiety, which prevents us from delving deeper into this critical aspect.

This study found a high prevalence of anxiety in Brazilian children compared to previous research (7). In the present study, the prevalence of anxiety among children, based on a CAQ score of ≥9 and an NRS score of ≥8, was 24.9% (*n* = 226) and 34.9% (*n* = 316), respectively. These results endorse a systematic review and meta-analysis that shows that anxiety symptoms were more prevalent in the second wave of COVID-19 than in the first wave of COVID-19 (5). The results underscore the significance of consistently examining anxiety in children within the context of the COVID-19 pandemic. In this study, we replicated a prior investigation, a notable advantage. Furthermore, the data collection period might have coincided with the peak of the pandemic. Brazil is a continental country. At the same time, it has more developed cities and regions, such as the South and Southeast regions, and others with much lower development rates, such as the North and Northeast regions, which exacerbates social inequalities. Despite that, we did not find an association between children’s anxiety and regions of Brazil.

According to the CAQ and NRS instruments, children’s anxiety was significantly associated with chronic diseases or disabilities. A systematic review, with a total of 116 articles representing more than 127,923 children and adolescents, shows that neurodiverse children and adolescents, and those with pre-existing mental illness have experienced higher levels of psychological distress, depression, anxiety, and behavior problems since the onset of the pandemic. Similarly, those with chronic physical health (including respiratory) conditions also experienced more severe mental health impacts than those without them (19). A cross-sectional study conducted in Brazil with 355 adolescents with different chronic conditions and 111 healthy adolescents, aged 10 to 18 years old, between July and October 2020, showed no statistical difference in the Strengths and Difficulties Questionnaire (SDQ) total score in patients with chronic disease and the control, 30% vs. 31%, *p* = 0.775, respectively. These findings differ from this study where chronic disease and disability increased anxiety one year after the onset of the pandemic (22).

Chronic diseases are health problems that persist over time, require continuous management in life, and could have periods of clinical instability, leading to hospitalizations and complex care. The most common chronic disease reported by parents was asthma/bronchitis, which could be a complication for a child with a COVID-19 diagnosis. Consequently, greater attention should be paid to these children and their families in the community, schools, primary care, and hospitals. During the COVID-19 pandemic, managing chronic diseases in childhood became more complicated for families and health professionals, as highlighted in different countries. A study in France revealed that 34.7% of children and adolescents with ADHD had or experienced marked deterioration in wellbeing, as shown by attitudes toward opposition or audacity, emotion, internal outbursts, sleep problems, and anxiety (23). A Dutch study of 75 children (median age = 10.5 years) with severe obesity showed that anxiety related to COVID-19 occurred in 32% (24). The possibility of health problems among children with chronic diseases justifies the higher anxiety levels among family members, which may, in turn, influence the child. This outcome was highlighted in an editorial about the challenges posed by COVID-19 to children with cancer at the beginning of the pandemic (25). In Germany, a survey of with 210 parents of children with rare congenital surgical diseases (anorectal malformations, biliary atresia, congenital diaphragmatic hernia, esophageal atresia, or Hirschsprung’s disease) and 88 parents of children without rare diseases. Results of these studies showed that the former reported severe psychosocial impairment among themselves and their children during the COVID-19 pandemic (26).

Finally, the children school status was not associated with the CAQ or NRS. However, notably, 3.5% of the children attended presential class. Moreover, research shows an increase in mental health symptoms because of school closure (4, 5, 27). A systematic review and meta-analysis included 868,634 children and adolescents (≤ 19 years) pre-pandemic and 807,480 during the COVID-19 pandemic in Europe. It compares the depression symptoms during the pre-pandemic vs. pandemic periods, showing that school closures during the pandemic resulted in a considerable increase in depression symptoms (27). Another systematic review and meta-analysis included 26 publications (*n* = 15,038 pre-pandemic, *n* = 13,041 during pandemic) in Europe, revealed during the pandemic a significant reduction in total physical activity and moderate-to-vigorous physical activity, corresponding to a decrease of 12 min per day. A decline in physical activity and a simultaneous increase in mental health disorders may have contributed to a general worsening of the health status of children and adolescents during the COVID-19 pandemic (28).

Hence, school staff and health professionals must increase awareness regarding feelings of anxiety in children for early prevention of m entail illness, especially in children with chronic diseases or disabilities. To fully grasp how Brazilian children were affected by the pandemic, each federal state in Brazil should understand and fulfil the children’s specific necessities required and conduct additional long-term studies. Moreover, new studies should evaluate anxiety among children in middle- and low-income countries such as Brazil and the return to school after absence due to school closures.

### Limitations

Some limitations of this study should be noted. The main limitation is the sample and data collection. We employed a non-probability sampling method, and the data collection was affected by participant selection bias. The data collection was conducted online, with participation restricted to those with access to resources (computers, telephones, and Wi-Fi) and those with the skills to read and respond. Then, more vulnerable adolescents without internet access were not represented in our study, which highlights the need for further studies that include this population.

Additionally, parents with more than one child in the house who answered multiple surveys were not excluded. We could not verify whether the research participation location was a quiet environment free from distractions. Furthermore, we did not investigate the guardians’ income levels to associate with children’s anxiety. Consequently, caution should be exercised when generalizing the data from the current study. Thus, future studies may adopt face-to-face interviews as a suitable data collection method, with face-to-face data collection.

### Implications for nursing practice

Finally, we hope that our research contributes to knowledge that can guide public policies and contribute to the 17 sustainable goals aimed at ensuring that no child is left behind. Goal 3 describes good health and wellbeing, where fewer children are ill and all children have the right to feel physically and mentally healthy. This target, to be implemented by 2030, promotes preventive measures to prevent and improve children’s mental health and wellbeing.

## Conclusion

The prevalence of anxiety among children a year after the beginning of the COVID-19 pandemic was 24.9% (*n* = 226) and 34.9% (*n* = 316), according to the CAQ and NRS, respectively. Higher anxiety levels were found to be associated with chronic disease or disability in children.

## Data availability statement

The raw data supporting the conclusions of this article will be made available by the authors, without undue reservation.

## Ethics statement

The studies involving humans were approved by the Research Ethics Committee of Brazil (CAAE: 30547320.0.0000.0008 and Opinion nº 4.128.847). The studies were conducted in accordance with the local legislation and institutional requirements. Written informed consent for participation in this study was provided by the participants’ legal guardians/next of kin.

## Author contributions

MA: Data curation, Funding acquisition, Investigation, Project administration, Writing – review & editing. TJ: Investigation, Writing – original draft. PF: Formal analysis, Methodology, Writing – review & editing. GA: Investigation, Writing – original draft. PO-C: Formal analysis, Methodology, Writing – review & editing. MB: Writing – review & editing. MJN: Writing – review & editing. SN: Conceptualization, Methodology, Writing – review & editing.
