# Supplementary material for: Anxiety among children a year after the onset of the COVID-19 pandemic: a Brazilian cross-sectional online survey
Source: Front Public Health. 2024 Jun 19;12:1372853. doi: 10.3389/fpubh.2024.1372853 (PMC11220266; doi:10.3389/fpubh.2024.1372853)
Supplement: Supplementary file 2 [file Table_2.docx]

**Supplementary Material**

**
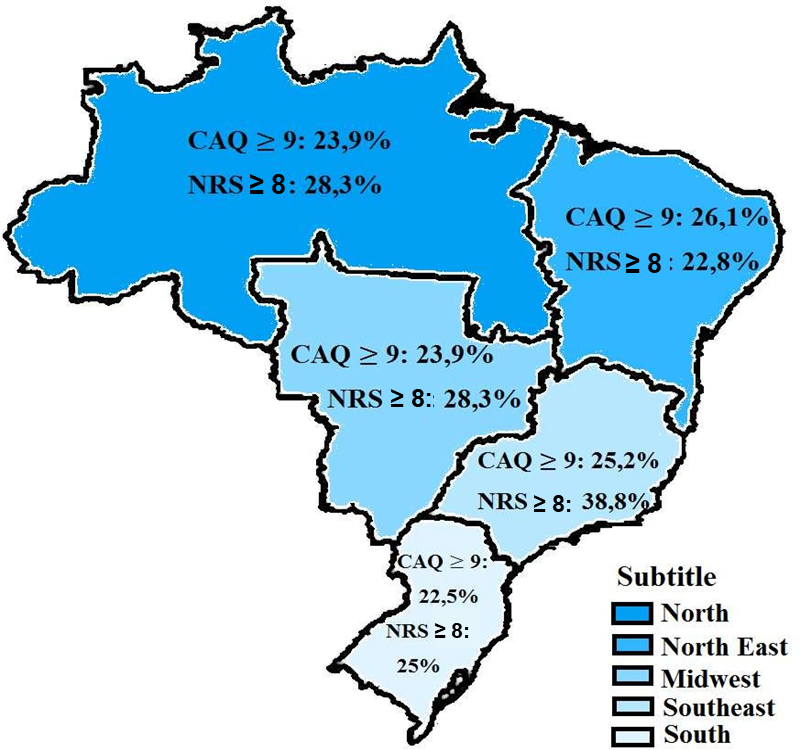
**

Figure 1. Prevalence of child anxiety according to Brazilian region (CAQ score ≥ 9;

NRS score **≥** 8 ). n=906, Botucatu, SP, Brasil, 2020
