# Supplementary material for: Anxiety among children a year after the onset of the COVID-19 pandemic: a Brazilian cross-sectional online survey
Source: Front Public Health. 2024 Jun 19;12:1372853. doi: 10.3389/fpubh.2024.1372853 (PMC11220266; doi:10.3389/fpubh.2024.1372853)
Supplement: Supplementary file 3 [file Table_3.docx]

**Supplementary Material**

Sociodemographic and COVID-19-related Characteristics of the Children and Their Guardians. n=906, Botucatu, SP, Brasil, 2020

| Variable | N | % |
| --- | --- | --- |
| Child’s gender |  |  |
| Female | 483 | 53.3 |
| Male | 422 | 46.6 |
| Non-binary | 01 | 0.1 |
| Mean age ± SD (years) | 8.79 ± 2.05 |  |
| Education |  |  |
| Home school | 726 | 80.1 |
| Hybrid classes | 105 | 11.6 |
| Presential school | 32 | 3.5 |
| On Vacation | 26 | 2.9 |
| Missing School | 17 | 1.9 |
| Public school (yes) | 299 | 33.0 |
| Had a pet (yes) | 641 | 70.8 |
| Lived in house | 704 | 77.7 |
| Hours in social distancing (mean ± SD) | 21.52 ± 5.37 |  |
| CAQ median (range) | 7.15 (4-12) |  |
| NRS median (range) | 5.98 (0-10) |  |
| Guardians’ mean age ± SD (years) | 38.45±8.07 |  |
| Home office | 416 | 45.9 |
| Income decreased (yes) | 432 | 47.7 |
| COVID-19 health professionals (yes) | 204 | 22.5 |
| Take care of the children* |  |  |
| Mother | 465 | 51.3 |
| Father | 55 | 6.1 |
| Both | 208 | 23.0 |
| Other family member | 127 | 14.0 |
| Babysitter | 47 | 5.2 |
| Alone | 04 | 0.4 |
| Schooling |  |  |
| Postgraduate | 449 | 49.6 |
| Graduate | 274 | 30.2 |
| High school | 146 | 16.1 |
| Elementary school | 29 | 3.2 |
| Median number of persons in house (range) | 4.21 (2-12) |  |
| Median number of children in house (range) | 1.74 (2-5) |  |
| COVID-19 suspected in family house (yes) | 472 | 52.1 |
| Confirmed COVID-19 (yes) | 240 | 26.5 |
| Region of Brazil |  |  |
| Southeast | 642 | 70.9 |
| Northeast | 92 | 10.2 |
| South | 80 | 8.8 |
| North | 46 | 5.1 |
| Midwest | 46 | 5.1 |
| Child’s perceived comprehension |  |  |
| A lot | 512 | 56.5 |
| Some | 230 | 25.4 |
| A little | 154 | 17.0 |
| None | 10 | 1.1 |

CAQ, children’s anxiety questionnaire; NRS, numerical rating scale; SD, standard deviation

*Person who took care of the children
